# Higher Medical Education

**Published:** 1894-01

**Authors:** 


### "HIGHER MEDICAL EDUCATION."

In pursuance of the policy recently announced in the resolution to be presented to the American Medical College Association, the Trustees and Faculty of Rush Medical College, have decided to require four years attendance at college from students who begin the study of medicine this year with a view to graduation in 1898; however, those who have already studied medicine one year or more with a preceptor, so that the four years of study, already required, will be completed before July, 1897, may graduate after three courses of lectures as heretofore. To encourage proper preliminary study, graduates in Arts and Sciences from high grade colleges, and graduates in Pharmacy and Dentistry from colleges requiring a proper amount of study and two full courses of lectures will, until further notice, be allowed to graduate after an attendance on only three courses of lectures.

